# “Characterization of ELEKTA SRS cone collimator using high spatial resolution monolithic silicon detector array”

**DOI:** 10.1002/acm2.12345

**Published:** 2018-05-22

**Authors:** Khalsa Al Shukaili, Stéphanie Corde, Marco Petasecca, Vladimir Pereveratylo, Michael Lerch, Michael Jackson, Anatoly Rosenfeld

**Affiliations:** ^1^ Centre for Medical Radiation Physics University of Wollongong Wollongong NSW Australia; ^2^ National Oncology Centre Royal Hospital Muscat Oman; ^3^ Illawarra Health and Medical Research Institute Wollongong NSW Australia; ^4^ Nelune Comprehensive Cancer Centre Prince of Wales Hospital Randwick NSW Australia; ^5^ SPA BIT Ukraine

**Keywords:** stereotactic radiotherapy, small field dosimetry, silicon detector, IBA SFD, EBT3

## Abstract

**Purpose:**

To investigate the accuracy of the dosimetry of radiation fields produced by small ELEKTA cone collimators used for stereotactic radiosurgery treatments (SRS) using commercially available detectors EBT3 Gafchromic^TM^ film, IBA Stereotactic diode (SFD), and the recently developed detector DUO, which is a monolithic silicon orthogonal linear diode array detector.

**Methods:**

These three detectors were used for the measurement of beam profiles, output factors, and percentage depth dose for SRS cone collimators with cone sizes ranging from 5 to 50 mm diameter. The measurements were performed at 10 cm depth and 90 cm SSD.

**Results:**

The SRS cone beam profiles measured with DUO, EBT3 film, and IBA SFD agreed well, results being in agreement within ±0.5 mm in the FWHM, and ±0.7 mm in the penumbra region. The output factor measured by DUO with 0.5 mm air gap above agrees within ±1% with EBT3. The OF measured by IBA SFD (corrected for the over‐response) agreed with both EBT3 and DUO within ±2%. All three detectors agree within ±2% for PDD measurements for all SRS cones.

**Conclusions:**

The characteristics of the ELEKTA SRS cone collimator have been evaluated by using a monolithic silicon high spatial resolution detector DUO, EBT3, and IBA SFD diode. The DUO detector is suitable for fast real‐time quality assurance dosimetry in small radiation fields typical for SRS/SRT. This has been demonstrated by its good agreement of measured doses with EBT 3 films.

## 1 INTRODUCTION

Stereotactic Radiosurgery (SRS) is an advanced radiotherapy treatment technique that is characterized by the delivery of high radiation dose to small volume to maximize the dose to kill the lesion, while minimizing the dose to the normal surrounding tissue. Hence, it requires very high accuracy and high conformity. One of the main issues with SRS dosimetry is the use of small fields either by using a micro‐MLC or a circular cone collimator. Small field dosimetry is known to be complex due to the existence of lateral charge disequilibrium, source occlusion, and volumetric effect if the size of the detector is comparable to the field size.^1^ Dosimetry protocols such as IAEA TRS‐398^2^ and AAPM TG‐51^3^ provide guidelines for traditional radiation fields (from 3 × 3 cm^2^ to 40 × 40 cm^2^), but are not designed for small field dosimetry. By extending the use of these codes for small field dosimetry, some researchers found a difference in the output factors by up to 30% between different detectors.^4^, ^5^, ^6^

For accurate and precise dosimetry of small field techniques, many groups have been working to get new protocols for MV small field dosimetry, which recommends specific requirements of detectors and use of different strategies.^7^, ^8^, ^9^, ^10^ Nowadays, there are a number of commercially available detectors for small field dosimetry, each having some characteristic that makes it suitable for some small field measurements. However, there is no single ideal detector that fulfills all the required characteristics for SRS dosimetry until now. Previously, many researchers suggested the use of different small size detectors and compare between them to overcome the drawbacks of each detector, to get the full characteristics of small beams.^11^

Different groups are working these days on silicon array detectors^12^, ^13^, ^14^; taking advantage of their excellent spatial resolution and small size compared with ion chambers, their real‐time measurements compared with EBT3 and TLDs, and their high sensitivity compared with ion chambers and EBT3. In addition, they are less expensive than diamond detectors and have reasonable uniformity and are more practical compared with gel dosimetry. Diodes work without external bias and they provide almost energy independence of mass collision stopping power ratios of silicon to water for electrons for clinical use in the range from 4 to 20 MeV.^15^

However, silicon detectors have some limitations, which need to be characterized to derive appropriate correction factors or to minimize these effects.^15^ The sensitivity of diodes is increased by increasing the instantaneous dose per pulse. This effect can be significantly minimized by selecting p‐type, preirradiation by large electron doses, or using heavy platinum doping or epitaxial guarded silicon diode.^16^, ^17^, ^18^ Another factor that can affect silicon diodes is the temperature, which could affect the level of recombination, and hence the sensitivity of the detector in a linear correlation. This variation in the sensitivity can be canceled by the preirradiation of the diode to high dose.^19^, ^20^ The energy dependence of a silicon diode at low photon energy (<200 keV) is related to the geometry and material surrounding the diode and diode material, which usually have higher atomic number when compared with water. Therefore, each diode should be selected with caution depending on the energy range it was designed for. Previous studies showed that the energy dependence increased as the thickness of the buildup material increased.^21^ However, for small field dosimetry such as SRS, diodes are used without shielding (almost no buildup material).^22^ In addition, it has been reported that silicon diodes have angular dependence when they are used in rotational beam measurements. This depends on their design and packaging. Several research groups have studied the angular dependence of diodes and found an over‐response up to 30% for 6 MV photon beams at 90° ± 10° and 270° ± 10°.^23^, ^24^, ^25^ Using different techniques can eliminate the angular dependence, such as adding half pipe‐shaped boluses,^26^ filling air gaps with sheets of lucite and pieces of copper,^27^ or by applying angular correction factors.^28^ Recently, edgeless diodes were developed which, combined with a special drop in packaging in kapton tails, avoided high Z overlayers, and demonstrated angular independence in MV photon field.^29^

The silicon diode arrays due to their atomic composition is different to water and leading to beam perturbation effect due to difference of secondary electron energy fluence in water and silicon radiation sensitive volume of the detector, therefore violating the condition for CPE and breaking down Bragg–Gray cavity theory. The low‐energy part of the differential electron fluence in silicon is larger than in not perturbed water. This difference is governed by the density term in electron mass stopping power of the radiation sensitive volume of the detector relative to water and by extracameral material (packaging) of the detector and depends on difference of ionizing potentials of Si and water. This is why calibration of silicon detector in a large beam where CPE is established and difference in electron energy fluence in water and silicon radiation sensitive volume is negligible is not valid for small field. For more details reader can references to Andreo (2018).^30^

The performance of the detector can be improved by removing the high atomic number and density materials near the sensitive volume or by adding low atomic number and density materials around the sensitive volume to compensate the over‐response due to higher Z material than water.^31^, ^32^ To compensate the effect of volume averaging due to the large sensitive volume of detector, the deconvolution method could be used. However, this method is complicated if done manually and is not practical in clinical setting as large number of profiles require long postprocessing time.^15^ Hence, different scientific papers have focused on finding proper correction factors to minimize the over‐response of the different diodes by studying their response for different beam qualities, field sizes, and types of LINACs in comparison with EBT3 and Monte Carlo simulation.^4^, ^33^, ^34^, ^35^, ^36^, ^37^

The Center of Medical Radiation Physics (CMRP) at the University of Wollongong is specializing in development of different types of silicon detectors for radiotherapy dose verification applications. The purpose of this study is to characterize recently released ELEKTA circular SRS cone collimator by using the high spatial resolution monolithic silicon diode array, DUO, for relative dosimetry of small radiation fields and compare it with some available commercial detectors.

## 2 MATERIALS AND METHOD

### 2.A Source and LINAC system

All measurements were performed in the radiation oncology department of the Nelune Comprehensive Cancer Centre, Prince of Wales Hospital (Randwick, NSW, Australia) by using 6 MV flattened photon beam from an ELEKTA Axesse^TM^ linear accelerator with a retrofitted Agility head (Elekta AB, Stockholm, Sweden), adapted for stereotactic treatment by using an additional gantry mounted ELEKTA cone collimators system. The cone collimators diameter varied from 5 to 50 mm, every 2.5 mm up to 20 mm, and then every 5 mm. The X (MLC) and Y jaw positions were set as in Table 1, for all measurements with these circular cones.

**Table 1:** The field sizes used for each circular cone diameter

Circular cone diameter (mm)	Field sizes (mm)
5, 7.5, 10	30 × 30
12.5, 15, 17.5, 20	40 × 40
25, 30	50 × 50
35, 40, 50	60 × 60

### 2.B Detectors

#### 2.B.1 Stereotactic field diode

The IBA stereotactic field diode (SFD) (IBA dosimetry, Nuremberg, Germany) is a p‐type unshielded low‐resistivity silicon diode. It has active volume of 0.017 mm^3^ with 0.06 mm thickness and 0.6 mm diameter. Its sensitivity is 6 nC/Gy.^38^ Morales et al.^39^ had studied the dose rate dependence of SFD by measuring the PDD for field size 10 × 10 cm^2^ and compared it with the ionization chamber. The results showed an agreement within 0.5%. It was confirmed that IBA SFD is almost dose rate independent. However, many authors have published correction factors for SFD detectors to correct the over‐response due to the density, nonwater equivalency, and the volume averaging effects for different LINACs and different depths in water.^4^, ^10^, ^33^, ^34^, ^35^, ^36^ In this study, we have used the correction factors provided by IAEA‐AAPM TRS‐483 10 to overcome the over‐response of IBA SFD detector in the output factors (OF) measurements.

#### 2.B.2 Gafchromic^TM^ Films (EBT3)

The EBT3 film is comprised of a 27 μm active layer, sandwiched between two 120 μm matte polyester layers, which make it more robust in principle for use in water. It is used in conjunction with an Epson 10000 XL film scanner, which enables RGB multichannel analysis. The dose range of the EBT3 film is up to 10 Gy with the red color channel. The EBT3 film is dose rate independent, near tissue equivalent, and can be used in water phantoms.^40^ The spatial resolution of EBT3 is determined mostly by the scanner resolution.

#### 2.B.3 DUO detector

The DUO detector was designed and developed at the CMRP. DUO is made of two orthogonal axial monolithic silicon diode arrays of 505 pixels with a total size of 52 × 52 mm^2^. Each diode measures 0.04 mm in the direction of linear array axis, and 0.8 mm in the orthogonal direction, with a pitch of 0.2 mm to provide the required spatial resolution for measurements of the sharp fall off penumbra region. An air gap of 0.5 mm was introduced above the DUO detector to compensate the over‐response that results from the high density of silicon and extracameral components.^41^ The diodes are operating in a passive mode (no bias). The DUO detector is placed on a thin printed circuit board (PCB‐500 μm thick), and connected to the fast data acquisition system (DAQ). The DAQ is based on a commercial analog front‐end ASIC named AFE0064 (Texas Instruments), which consists of 64 channels with each providing an analog differential output proportional to the charge accumulated in a capacitor during a determined time frame. The DAQ system contains eight AFE0064 chips, for 512 channels in total. The acquisition of the data is synchronized with the LINAC by using the pulse‐by‐pulse scope trigger available on the service panel. These are connected to a fully programmable gate array (FPGA) that used for a signal processing. The digital data then sent to the computer via USB cable for analysis.

## 3 MEASUREMENTS

### 3.A Beam profiles and output factors

To characterize the performance of the DUO detector in the radiation field collimated by the ELEKTA SRS circular cones, profiles from the cones of sizes 5, 7.5, 10, 12.5, 15, 17.5, 20, 25, 30, 35, 40, and 50 mm were measured at 10 cm depth in a Solid Water^®^ phantom (GammaX, Middleton, WI, USA). The phantom size is 30 × 30 cm^2^ to provide the proper scattering conditions and 10 cm slabs were used for backscatter. The DUO detector was irradiated with 100 MU at 90 cm SSD by 6 MV photon beam, recording three sets of measurements for each settings. The beam profiles were measured in both X and Y profiles simultaneously. These data were compared with EBT3 film data that were taken under the exact same experimental conditions. IBA SFD was used to measure the beam profiles in a water phantom instead of the Solid Water^®^ phantom.

The dose profile measured by DUO was in steps of 0.2 mm, corresponding to the pixels pitch of DUO, while EBT3 measured dose points every 0.35 mm that is determined by the resolution of the scanner 72 dpi. The SFD diode data were obtained by scanning in a water phantom with steps of 0.3 mm. The profiles measured by EBT3 films and the IBA SFD diode were interpolated by using MATLAB^TB^ software to 0.2 mm by using the cubic shape preserving function. The FWHM and penumbra width were calculated by using MATLAB^TB^ software.

The OFs measured by DUO were calculated as the ratio of the dose response at the central pixels of the DUO detector for each SRS cone size at 10 cm depth to the dose response at the same central pixels for the 50 mm SRS cone size at the same depth. These OF are compared with the OF taken by IBA SFD and EBT3 films. For the IBA SFD diode, the OF were calculated using the correction factors.

### 3.B Percentage depth dose

**Figure 1:**
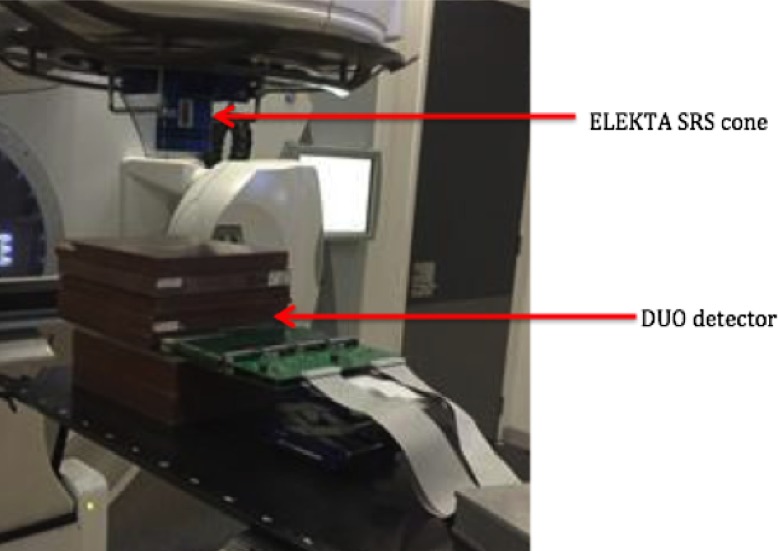
Setup for PDD measurements using DUO detector with Solid Water^®^ slabs for Elekta SRS circular cone collimator at 90 cm SSD.

To investigate the performance of DUO at each depth for different SRS cone sizes, the percentage depth dose (PDD) was measured in Solid Water^®^ at 90 cm SSD (Fig. 1). Solid Water^®^ slabs were placed on the bottom (10 cm) for backscattering purposes. The PDD was measured for cone sizes from 5 to 40 mm at depths from 0.5 to 25 cm. The measurements were repeated three times for each setting to calculate the error from the standard deviation. Corresponding measurements were performed with EBT3 for comparison. IBA SFD was used also to measure the PDD in water phantom at 90 cm SSD, for all cone sizes. It should be noted that the PDD measured by DUO was corrected for the dose per pulse (DPP) variation as calculated before by Al Shukaili et al.^41^

### 3.C EBT3 film measurements

**Figure 2:**
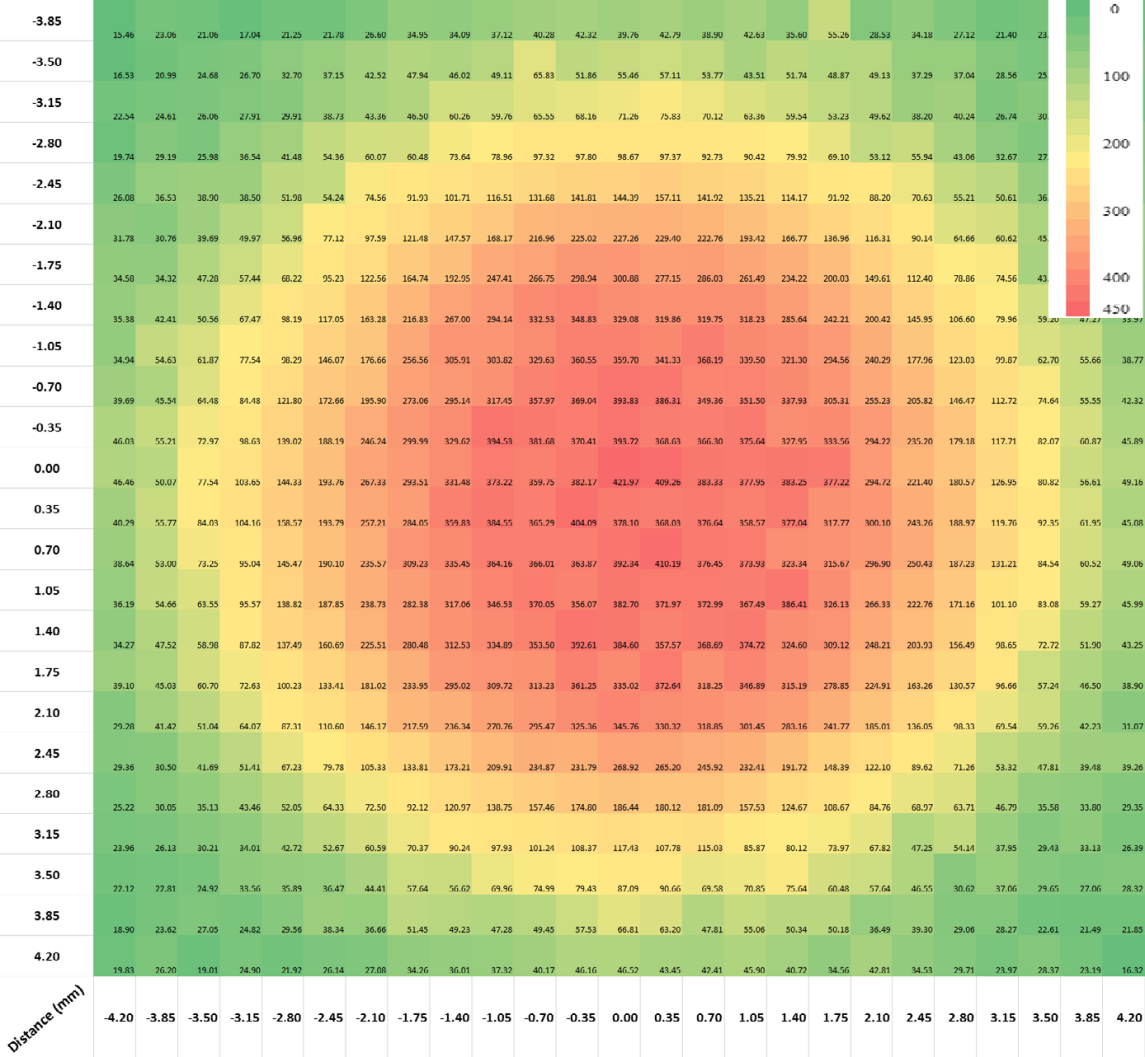
2D dose map for 5 mm cone diameter at 10 cm depth, (dose in cGy).

Gafchromic^TM^ EBT3 films were used to compare the measured beam profiles, OF, and PDD from SRS cones by the DUO detector. Ten EBT3 calibration films were cut into 3 × 3 cm^2^ to get the calibration curve, irradiated from 50 to 600 MU. The measurement films were cut into sections so that it is larger than each SRS cone size by 2 cm to measure the beam profiles and PDD. The films were prescanned and postscanned with an Epson scanner (10000 XL), where the postscan was performed 48 h after irradiation. The films were scanned in transmission mode using 48‐bit RGB color mode (no color corrections applied) with 72 dpi scan resolution. The films were placed at the center of the scanner by using a template to reduce the optical nonuniformity and scanned in one fixed direction to avoid film‐induced change in pixel values. For irradiation, the EBT3 films were placed between Solid Water^®^ slabs in an orientation perpendicular to the beam central axis (CAX) and were irradiated by 500 MU. The analysis was performed using MATLAB and pixel values in red channel were used to calculate the optical density. The dose calibration curve was fitted using a third‐order polynomial function. The dose maps were calculated using MATLAB and the center of the cone was determined to calculate the OF, PDD, and beam profiles in two dimensions. An example for cone 5 mm at 10 cm depth is presented in Fig. 2.

## 4 RESULTS

### 4.A Beam profiles

**Figure 3:**
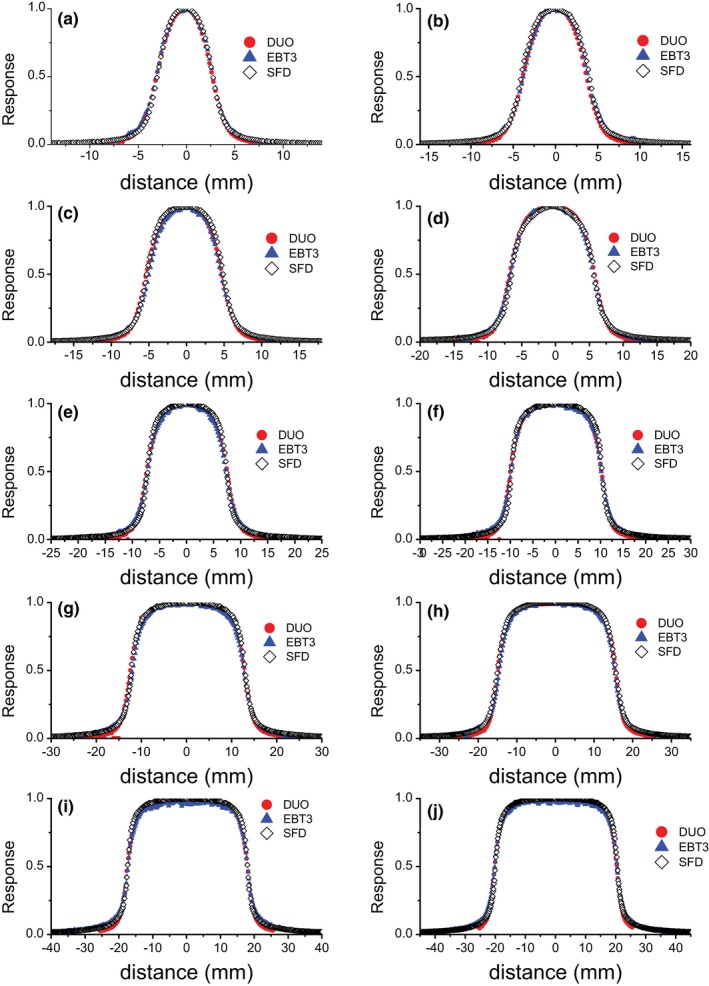
Cross‐plane profiles measured by DUO, EBT3, and SFD at 90 cm SSD, for cone diameters: (a) 5 mm, (b) 7.5 mm, (c) 10 mm, (d) 12.5 mm, (e) 15 mm, (f) 20 mm, (g) 25 mm, (h) 30 mm, (i) 35 mm, and (j) 40 mm.

Both cross‐plane and in‐plane beam profiles of all cone sizes were measured using DUO, SFD, and EBT3 films. The cross‐plane profiles of the radiation fields from SRS cones of different sizes were compared as shown in Fig. 3. The beam profiles were normalized to 1 at the center. In general, there is an agreement between the three detectors in both X and Y profiles.

The beam profile parameters (FWHM and penumbra width 20%–80%) were calculated for each cone size in the X and Y profiles for the three detectors as shown in Tables 2 and 3. The 20%–80% penumbra width is calculated as the average between the 20%–80% ascending and descending parts on the beam profiles. There is a good agreement among DUO, EBT3 films, and IBA SFD within ±0.5 mm for FWHM. The 20%–80% penumbra widths showed a good agreement within ±0.7 mm for all cone sizes. By comparing the profile parameters between X profiles and Y profiles, there were differences up to 0.3, 0.6, and 0.8 mm in FWHM measured by DUO, EBT3, and IBA SFD, respectively. In terms of the penumbra width, the differences between X profiles and Y profiles were high, up to 0.5, 0.6, and 1.0 mm, measured by DUO, EBT3, and IBA SFD, respectively.

**Table 2:** FWHM measured by DUO, EBT3, and SFD in both X profiles and Y profiles

Cone	FWHM (mm)						Differences (mm)			
	X profile			Y profile			DUO/EBT3		DUO/SFD	
mm	DUO	EBT3	SFD	DUO	EBT3	SFD	X profile	Y profile	X profile	Y profile
5	6.13	6.20	6.00	5.87	5.80	5.80	−0.07	0.07	0.13	0.07
7.5	7.60	8.00	7.80	7.53	7.60	7.40	−0.40	−0.07	−0.20	0.13
10	10.16	10.40	10.20	10.16	10.00	9.80	−0.24	0.16	−0.04	0.36
12.5	12.85	13.00	12.80	12.76	12.60	12.40	−0.15	0.16	0.05	0.36
15	15.05	14.80	15.40	14.79	14.80	14.60	0.25	−0.01	−0.35	0.19
20	20.25	20.40	20.60	20.19	19.80	20.60	−0.15	0.39	−0.35	−0.41
25	25.51	25.40	25.60	25.68	25.40	25.40	0.11	0.28	−0.09	0.28
30	30.71	30.20	30.80	30.68	30.40	30.80	0.51	0.28	−0.09	−0.12
35	35.57	35.20	36.00	35.68	35.80	35.80	0.37	−0.12	−0.43	−0.12
40	40.83	40.40	41.00	40.91	40.60	40.80	0.43	0.31	−0.17	0.11

**Table 3:** Penumbra measured by DUO, EBT3, and SFD in both X profiles and Y profiles

Cone	Penumbra (mm)						Differences (mm)			
	X profile			Y profile			DUO/EBT3		DUO/SFD	
mm	DUO	EBT3	SFD	DUO	EBT3	SFD	X profile	Y profile	X profile	Y profile
5	2.04	2.00	1.60	1.81	1.80	1.80	0.04	0.01	0.44	0.01
7.5	2.50	2.60	2.20	2.27	2.00	1.90	−0.10	0.27	0.30	0.37
10	2.50	2.80	2.20	2.50	2.20	1.80	−0.30	0.30	0.30	0.70
12.5	2.96	3.00	2.60	2.47	2.60	2.20	−0.04	−0.13	0.36	0.27
15	2.96	3.20	2.50	2.50	2.80	2.20	−0.24	−0.30	0.46	0.30
20	2.50	3.20	2.80	2.73	3.20	2.20	−0.70	−0.47	−0.30	0.53
25	3.19	3.40	3.00	2.96	3.20	3.00	−0.21	−0.22	0.19	−0.04
30	3.42	3.60	3.30	3.19	3.20	3.60	−0.18	−0.01	0.12	−0.41
35	3.65	3.80	3.60	3.19	3.80	2.60	−0.15	−0.61	0.05	0.59
40	3.65	4.00	3.30	3.19	3.80	3.10	−0.35	−0.61	0.35	0.09

**Table 4:** The differences in FWHM and penumbra between X profiles and Y profiles measured by DUO, EBT3, and SFD, calculated as (X‐profile–Y‐profile)

Cone	FWHM (mm)			Penumbra (mm)		
mm	DUO	EBT3	SFD	DUO	EBT3	SFD
5	0.26	0.40	0.20	0.23	0.20	0.20
7.5	0.07	0.40	0.40	0.23	0.60	0.30
10	0.00	0.40	0.40	0.00	0.60	0.40
12.5	0.09	0.40	0.40	0.49	0.40	0.40
15	0.26	0.00	0.80	0.46	0.40	0.30
20	0.06	0.60	0.00	0.23	0.00	0.60
25	−0.17	0.00	0.20	0.23	0.20	0.00
30	0.03	−0.20	0.00	0.23	0.40	0.30
35	−0.11	−0.60	0.20	0.46	0.00	1.00
40	−0.08	−0.20	0.20	0.46	0.20	0.20

### 4.B Output factors

**Figure 4:**
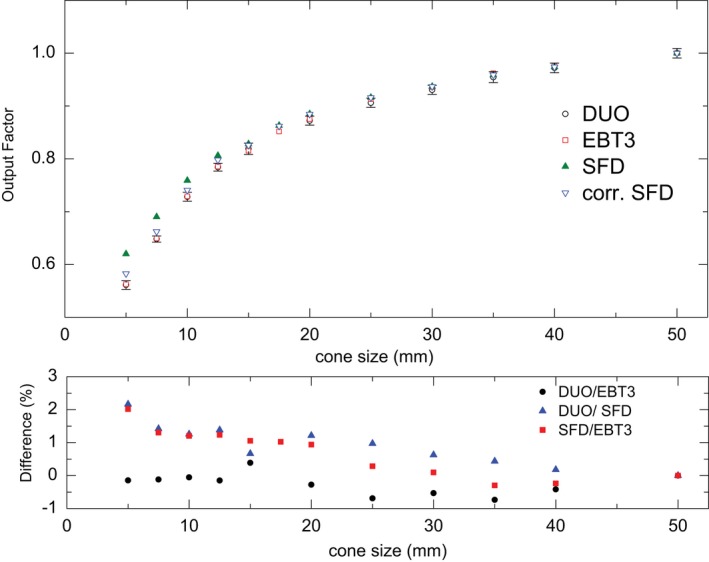
The output factors for cones of different size normalized to cone 50 mm diameter as measured by DUO, EBT3, and SFD, lower panel: the DUO/EBT3, DUO/corr. SFD, and corr. SFD/EBT3 percentage difference (%).

Figure 4 shows the OF for different cone sizes normalized to the 50 mm cone size, as measured by DUO, IBA SFD, and EBT3 films, and their percentage local deviations. The error on the DUO measurements was calculated as 2 standard deviations of three DUO measurements under the same setting (they are not clear in the figure because they are very small). The results show a good agreement between DUO and EBT3 within ±0.7% for all cone sizes. The IBA SFD shows an over‐response up to 6% before applying the correction factors. After applying the over‐response correction factors for IBA SFD, the average difference between IBA SFD with DUO and EBT3 output factors was within 1% with the maximum difference of 2.1% for 5 mm cone.

### 4.C Percentage depth dose

**Figure 5:**
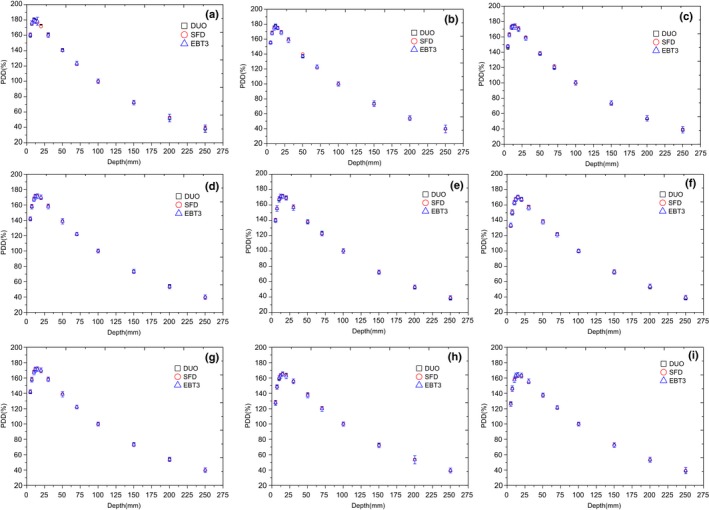
Percentage depth dose for different cone diameters measured by DUO, SFD, and EBT3 at SSD 90 cm for cone diameters: (a) 5 mm, (b) 7.5 mm, (c) 10 mm, (d) 12.5 mm, (e) 15 mm, (f) 20 mm, (g) 25 mm, (h) 30 mm, and (i) 40 mm.

**Figure 6:**
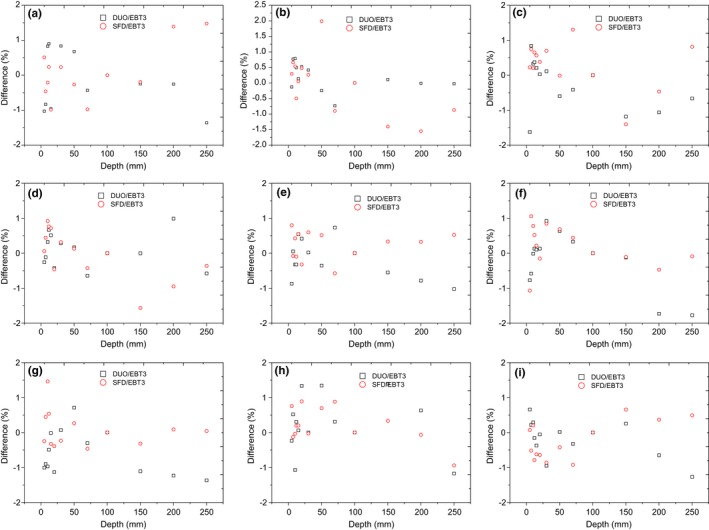
The differences in the PDD between DUO/EBT3 and SFD/EBT3; for cone diameters: (a) 5 mm, (b) 7.5 mm, (c) 10 mm, (d) 12.5 mm, (e) 15 mm, (f)20 mm, (g) 25 mm, (h) 30 mm, and (i) 40 mm.

Figures 5 and 6 show the PDD measured by using DUO, SFD, and EBT3 films for cone sizes of 5, 7.5, 10, 12.5, 15, 20, 25, 30, and 40 mm at depths up to 25 cm, and the percentage deviations between the detectors. The depth of maximum dose (d_max_) was different for each cone size as measured by DUO, IBA SFD, and EBT3, therefore the data were normalized at 10 cm depth to compare between them. Actually, the depth of maximum dose is around 12.2 mm for 5 mm cone size and it increases to around 15.2 mm for 40 mm cone size. The error in the DUO measurements was calculated as 2 standard deviations from three sets of measurements at the same setup (error bars are not clear in the figure as they are very small). The error bar in the EBT3 measurements represents 2 standard deviations of the central dose area from the average dose for each cone diameter.

The comparison of PDD measured by the three detectors for all cone sizes showed overall average agreement of ±0.5%; with maximum differences within ±2% for DUO/EBT3 and SFD/EBT3.

## 5 DISCUSSION

In this study, three detectors were used to measure the beam profiles, output factors, and PDD for ELEKTA SRS cone collimators. One of these detectors is water equivalent and has sufficient spatial resolution for small field dosimetry (EBT3) and two are diodes (DUO 2D high spatial resolution monolithic diode array and a single diode IBA SFD), which required correction for the nonwater equivalence and/or volume averaging effects.

### 5.A Beam profiles

By comparing the beam profiles of the SRS cone collimators, DUO shows good agreement with the EBT3 films in the “in‐field” area, but slightly lower dose in the “out‐field” area for all the cone sizes. This could be due to the dose rate dependence of DUO for very low dose rate.

**Figure 7:**
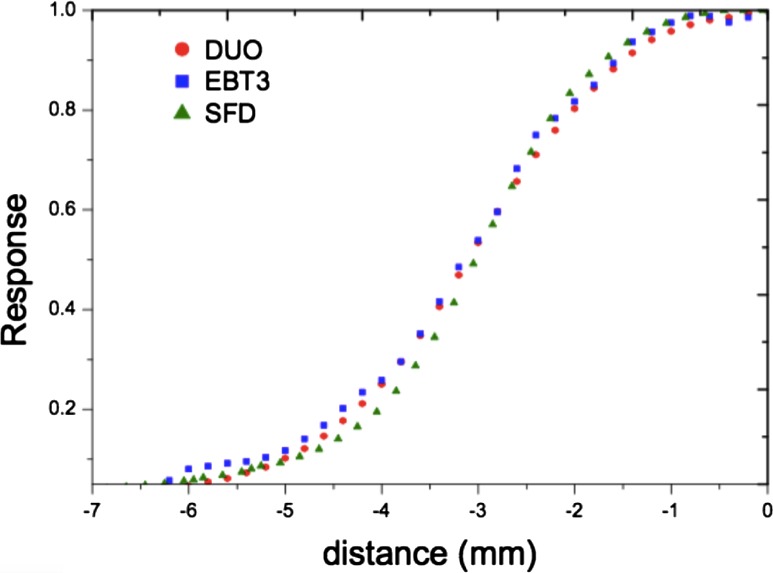
Penumbra comparison between DUO, SFD, and EBT3 for SRS cone diameter: 5 mm.

The normalized response measured by SFD shows higher drop off in the penumbra region than DUO and EBT3 for cone sizes smaller than 20 mm, as shown in Fig. 7. Taylor et al.^42^ also found this. The same behavior was found in the comparison of Y profiles. This could be due to the large sensitive volume and high Z extracameral material that has been explained in more detail by Benmakhlouf and Andreo.^43^ It could be also improved by using the deconvolution method, which has been developed to obtain beam profiles independent of the detector size.^44^ The results depend on both the pitch size (and spatial sampling in case of SFD) and the size of the sensitive volume of the detector. DUO shows good agreement in comparison with EBT3 and IBA SFD diode in both FWHM and penumbra width, for both X and Y profiles.

It was observed that penumbra measured in X/Y directions for cones is less than for Varian linac equivalent square fields as presented in Ref. 41. Smaller penumbra for cones in comparison with penumbra for equivalent square filed is partially due to cones closer to the phantom surface and different geometry and scattering in cones in comparison with jaws. This relative difference is decreasing with field increasing as expected.

However, all three detectors show differences between X and Y profiles in terms of FWHM and 20%–80% penumbra widths (Tables 2 and 3). X‐profile parameters were higher than Y‐profile parameters, which could be due to the elliptical‐shaped focal spot of the ELEKTA Axesse linac source. Different groups have studied the shape and size of the x‐ray source and found that it depends on the linac model, with mostly elliptical shape.^45^, ^46^, ^47^ The observed eliptical shape focal spot for Elekta was not observed for Varian Linac.^41^

### 5.B Output factors

A number of studies reported that diode over‐response in small fields is due to the density of silicon and the extracameral materials surrounding the detector. However, it has been recently clarified that electron density, rather than density as a fundamental characteristic of material, is driving the diode response through density effect in mass stopping power of electrons and ionization potential of silicon.^42^ In this study, the DUO silicon detector was corrected by using a 0.5 mm air gap above detector as studied earlier.^41^ This correction provides an overall agreement in the output factors for cone sizes from 5 to 50 mm, between DUO and EBT3 within ±0.7%.

IBA SFD diode was corrected by using the correction factors provided by TRS‐483.^10^ This led to better agreement in the OF measurements between IBA SFD and EBT3, where the difference reduced from 5.7% to 2% for the 5 mm cone size. The average agreement in the OF for all cone sizes is about ±0.8% after applying the correction factors for the IBA SFD.

Recently published AAPM practice guidelines recommend SRS‐SBRT annual QA for the OF and the tolerance is ±2% from the baseline for >1.0 cm apertures, and ±5% from the baseline for ≤1.0 cm apertures.^48^ Our results showed a good agreement between the three detectors indicating that DUO could be a suitable candidate for stereotactic cones regular QA. Its practicality and online reading would make it the preferred option over the other two detectors in clinical settings.

### 5.C Percentage depth dose

In the PDD measurements, the depth of maximum dose (d_max_) was changed as a function of cone diameter and it was difficult to detect the exact d_max_ as it depends on the available phantom thickness and the resolution of the detector. Therefore, all PDD measurements were normalized to 100 mm depth.

The comparison of PDDs between DUO, EBT3, and IBA SFD shows an agreement within ±2% for small cone sizes up to 20 mm, and then the agreement increased to ±1.5% for the larger cones.

## 6 CONCLUSION

It was recommended in the IPEM report 103 to avoid the use of ion chambers for the small beam profile measurements due to various issues such as their large volume, which causes volume averaging effects, therefore artificial broadening in the penumbra region, and increasing the FWHM of the measured beam profile.^9^ In addition, the use of small air‐filled ionization chambers causes under‐response to the dose due to their low mass density of air.^49^ Thus, the use of Gafchromic^TM^ films and diodes was recommended. To facilitate small field dosimetry and propose an alternative to time‐consuming films and single diode water tank measurements, CMRP designed a monolithic silicon detector DUO with 0.2 mm spatial resolution for SRS dosimetry. The effect of the high density of silicon on the output factor measurements was successfully compensated by introducing an air gap of 0.5 mm above it, as proposed by others for single diodes.^50^, ^51^

The circular SRS cones used with the ELEKTA LINAC were characterized in terms of the beam profiles, output factors, and PDD for cone diameters ranging from 5 to 50 mm and by using high spatial resolution detectors DUO, EBT3 films, and IBA SFD. The results showed that DUO agrees with EBT3 in terms of beam profiles and output factors. The good agreement between DUO, EBT3, and IBA SFD in the profiles shows a difference within ±0.5 mm in the FWHM and ±0.7 mm in the 20%–80% in the penumbra width. The output factors show very good agreement between DUO and EBT3 for all cone sizes within ±0.7%. IBA SFD detector agrees with the EBT3 and DUO measurements of output factors after applying the volume averaging correction factors, which shows an average agreement of ±0.8%, with maximum difference about 2% for 5 mm cone. In the percentage depth dose curves, there is a good agreement among DUO, SFD, and EBT3 for all depths of all measured cone diameters; with average difference within ±0.5% and maximum difference within ±2%.

In conclusion, ELEKTA SRS cones have been characterized by using three high spatial resolution detectors, two are commercially available and one is designed by CMRP at UOW. DUO is a suitable detector for fast SRS/SRT dosimetry as it has excellent resolution (0.2 mm) in a direction of steepest dose gradient, on line data analysis, and it provides both X and Y profiles. The good agreement with EBT3 films measurements confirms its accurate and precise data for SRS/SRT measurements, which is the treatment modality where small‐field dosimetry is paramount, and DUO can be applied successfully.

## CONFLICTS OF INTEREST

The authors have no relevant conflicts of interest to disclose.
